# Identification and Determination of the Seminiferous Epithelium Stages and Spermatid Development in the Testis of Aceh Bull (*Bos indicus*)

**DOI:** 10.1155/2023/8848185

**Published:** 2023-09-26

**Authors:** Sri Wahyuni, Tongku Nizwan Siregar, Gholib Gholib, Arianto Saputra, Hafizuddin Hafizuddin, Hamny Sofyan, Muhammad Jalaluddin, Mulyadi Adam, Muslim Akmal

**Affiliations:** ^1^Laboratory of Anatomy, Faculty of Veterinary Medicine, Universitas Syiah Kuala, Banda Aceh, Indonesia; ^2^Laboratory of Reproduction, Faculty of Veterinary Medicine, Universitas Syiah Kuala, Banda Aceh, Indonesia; ^3^Laboratory of Physiology, Faculty of Veterinary Medicine, Universitas Syiah Kuala, Banda Aceh, Indonesia; ^4^Study Program of Veterinary Medicine Education, Faculty of Veterinary Medicine, Universitas Syiah Kuala, Banda Aceh, Indonesia; ^5^Laboratory of Histology, Faculty of Veterinary Medicine, Universitas Syiah Kuala, Banda Aceh, Indonesia

## Abstract

This study was conducted to describe the stages of seminiferous epithelium (SE), determine the relative frequency of the stages, and identify the steps of spermatid development during spermatogenesis in the testicular tissue of Aceh bull. Seven pairs of the testicular organs of Aceh bull *(Bos indicus)* were used and then processed in a histological manner for staining using haematoxylin and eosin (H&E) and periodic acid-Schiff-haematoxylin (PAS-H). The stages of seminiferous tubules were examined using a tubular morphology method while spermatid development was observed based on the acrosome formation during spermatid development. Eight stages (stages I to VIII) of SE were found in the testicular seminiferous tubules of Aceh bull. Furthermore, the percentage of the relative frequency of each stage was 25.48, 15.38, 12.92, 4.74, 14.97, 10.69, 10.74, and 5.08%, respectively, with the relative frequency of premeiotic, meiotic, and postmeiotic phases being 53.78, 4.74, and 41.48%, respectively. Spermatid development from round to elongated spermatids occurred in 14 steps. Steps 1 to 7 were observed in stage I, steps 8 and 9 in stage II, steps 10 and 11 in stage III, step 12 in stage IV, step 13 in stages V and VI, and step 14 in stages VII and VIII. These findings can be used as a basis for further studies, particularly in evaluating the abnormality of the cellular composition of the seminiferous tubule in each stage of spermatogenesis and also in determining daily sperm production in Aceh bull.

## 1. Introduction

Spermatogenesis is a crucial process for producing spermatozoa, in which information on the biological process in the Aceh bull *(Bos indicus)* testis has never been reported. In addition to completing data of reproductive aspect, this basic information is useful for determining daily sperm production in this bull to improve the quality of straw used in artificial insemination (AI) in Aceh cattle. Aceh cattle are one of the local beef cattle in Indonesia which are the results of crossbreeding between *Bos javanicus* and *Bos indicus* [1]. Aceh cattle are commonly found and farmed in Aceh Province, Indonesia, as a source of meat consumed by the Aceh community [2, 3]. Aceh cattle have been the pride of the Acehnese people for a long time. Since 2011, these cattle have been designated as one of the beef cattle breeds in Indonesia based on Minister of Agriculture Decree Number: 2907/Kpts/OT.140/6/2011 [4]. Designation of the status of Aceh cattle by the Indonesian government is very important because these cattle have several advantages such as good adaptability to Indonesia's tropical climate, especially in the Aceh region, resistance to tropical diseases, easy to maintain, and a good selling price [5–7]. Despite the cattle having several advantages, some problems related to the raising pattern of these cattle were found, including inbreeding due to the unavailability of data and arrangement of breeding, as well as the existence of negative selection in bulls which are good for fattening [8]. As a consequence, poor reproductive performance and low reproduction rates become common problems associated with these cattle [9]. Some measures, thus, need to be taken to increase the productivity and population of Aceh cattle, one of which is by inducing bulls to produce good quality spermatozoa that further use in supporting the AI program. The success of the AI program is expected to support an increase in Aceh cattle population especially in Aceh Province, Indonesia. This is in accordance with Sofyan et al. [10] that Aceh cattle are still in demand by breeders because they are relatively easy to maintain and are the main choice for consumers. In addition, Aceh beef has a good taste, the meat does not shrink easily after cooking, and the muscle fiber is finer than the brahman cross beef which is influenced by the size of the muscle fibers [11, 12].

As male gamete cells, spermatozoa are produced after going through a long and complex process called spermatogenesis [13]. Spermatogenesis is a finely regulated process of multiplication and differentiation of male germ cells to produce spermatozoa in the testicular seminiferous tubules [14]. Spermatozoa are a genetically unique male gamete that can fertilize an ovum and then produce offspring [15]. Normal spermatogenesis relies on Sertoli cells, which preserve cell junctions while providing nutrients for male germ cells [16]. Spermatogenesis is a highly coordinated process that involves two types of cell divisions called mitotic and two meiotic divisions. During spermatogenesis, spermatogonia which will become spermatozoa experience development in three important phases, namely, proliferative, meiotic, and differentiation phases [13] which involve different cell associations, namely, the germinal cells (spermatogonium, spermatocyte, and spermatid), Sertoli cells, and myoid cells [17]. Spermatogonia are located on the surface of the basement membrane of seminiferous tubules, followed by spermatocytes (primary and secondary), and spermatids (round and elongated) that develop progressively toward the lumen of seminiferous tubules [15]. The association of these cells is called stage, where each stage can be characterized by the changes of germinal cells and the spermatid nuclei shapes as well as the presence of meiotic divisions in a specific stage of seminiferous tubules. This characterization is known as the tubular morphology method, as described by Wahyuni et al. [18] in the testicular tissue of Javan muntjacs *(Muntiacus muntjak muntjak)*.

In addition to the tubular morphology methods, the identification of the stage can also be performed by observing the development of the acrosome and nuclei of spermatid during spermiogenesis that was applied previously by Dreef et al. [19] in the testis of long-tailed macaque *(Macaca fascicularis)* and Šturm et al. [20] in testicular tissue of rams *(Ovis aries)*. Observation of the morphological changes of the developing acrosome is the basis for identifying the steps of spermatid differentiation. The last phase of spermatogenesis involves spermatid elongation, namely, spermiogenesis, where the nucleus of a spermatid is remodelled by chromatin condensation [21]. After the acrosome has been formed, spermatid tails start to form from the cytoplasm of those cells that cover the flagella and dense mitochondria [19]. The acrosome is a special kind of organelle with a cap-like structure located in the anterior of the sperm head. This structure is derived from the Golgi apparatus and covers the head of spermatozoa [22]. The function of acrosome is to condense the cell nucleus that carries genetic materials and protect the spermatozoa while moving along the female reproductive tract during mating [23], and then undergo a series of cellular or physiological changes such as capacitation and acrosome reaction before they fertilize the ovum [15].

The tubular morphology method has been applied to many species, and eight stages of seminiferous tubules have been identified in the studies carried out in testis of goats *(Capra hircus)* [24, 25], donkeys *(Equus asinus)* and mules *(Equus mulus)* [26], landrace boars *(Sus scrofa domesticus)* [27], crioulo horses *(Equus ferus caballus)* [28], dogs (*Canis familiaris)* [29], and Javan muntjacs [18], whereas the second method has been applied to the testicular tissue of gerbils *(Meriones unguiculatus)* [30], long-tailed macaques [19], indigenous bull *(Bos indicus)* [31], mice *(Mus musculus)* [32], micromini pigs *(Sus scrofa domesticus)* [33], and rams [20]. However, the data regarding the stages of the seminiferous epithelium and spermatid development during spermatogenesis in the testis of Aceh bull have not been reported yet. Therefore, the objective of this study was to identify and determine the stages of SE using the tubular morphology method as well as to observe the development of the spermatids in the testicular tissue of Aceh bull based on the acrosome formation detection using the histochemical PAS staining.

## 2. Materials and Methods

### 2.1. Animals and Ethics Approval

This study was conducted based on the ethical approval for using animals from the Ethics Commission on the Use of Experimental Animals, Faculty of Veterinary Medicine, Universitas Syiah Kuala, with certificate number: 197/KEPH/V/2020.

### 2.2. Sample and Tissue Preparation

Testicular samples (dexter and sinister) were collected from seven adult Aceh bulls (aged 2-3 years) after being slaughtered in a local slaughterhouse in Banda Aceh City, Aceh Province, Indonesia. After the collection, testicular organs were immediately fixed using 10% neutral buffer formalin, followed by immersion in 70% ethanol. A small piece of testis was cut and then dehydrated through immersion in 70% to absolute ethanol, clearance in xylene, infiltration in liquid paraffin, and embedded to obtain a paraffin block. Subsequently, a paraffin block containing testicular samples was cut into 3 *μ*m thick sections by using a rotary microtome (Leica RM2235, Leica Biosystems, Nusslogh GmbH, Germany). Tissue sections were placed on the surface of slides and then stained with H&E and PAS staining.

### 2.3. Haematoxylin and Eosin (H&E) Staining

Prior to H&E staining, a deparaffinization process was carried out followed by rehydration of the testicular tissue slides. The deparafinization process was carried out by immersing all the slides in xylene solution and then followed by rehydrating the slides in absolute, 90%, 80%, and 70% ethanols. Then, the slides were rinsed with running water and followed by distilled water. After deparaffinization and rehydration, the slides were stained using Mayer's haematoxylin (Path Chem, BBC Biochemical, US), rinsed in running tap water, and continued in distillate water. Afterwards, slides were immersed in eosin Y solution (Path Chem, BBC Biochemical, US) for 5 minutes and subsequently rinsed in a graded serial of ethanol, starting from 70% to absolute, and clearance in xylene, mounted with Entellan® (Merck KGaA, Germany), and covered using coverslips.

### 2.4. Periodic Acid-Schiff-Haematoxylin (PAS-H) Staining

The procedure of PAS-H staining refers to Nakata et al. [32] with some modifications. Deparaffinized and rehydrated testicular tissue preparation (slides) were immersed in 0.5% periodic acid solution (Nacalai Tesque, Inc., Japan) for 10 minutes and then rinsed in running tap water and three times in distillate water. Furthermore, all slides were immersed in Schiff's reagent (Nacalai Tesque, Inc., Japan) for 15 minutes and then rinsed with sodium hydrogen sulfite water (composition: 10% NaHSO_3_, 1N HCl, and distillate water) three times for 3 minutes each, and then rinsed in running tap water, followed by distillate water. After rinsing in distillate water, sections were then stained using haematoxylin. After rinsing using running tap water and distillate water, sections were dehydrated, cleared, mounted, and covered using coverslips.

### 2.5. Identification of Seminiferous Epithelium Stages and Relative Frequency

In this present study, a tubular morphology method was applied to identify the stages of seminiferous tubules of Aceh bull observed based on the composition of spermatogenic cells (spermatogonium, spermatocyte, round spermatid, and elongated spermatid) and also the presence of Sertoli cell nuclei at each different stage. The characteristic of each stage (stages I–VIII) has been described in detail in other males by França et al. [34], Leal and Fran*ς*a [35], and Wahyuni et al. [18]. Furthermore, the characteristics of each stage were observed using a light microscope (Olympus CX31, Japan) and photographed using a camera (Sigma, Germany) connected to a light microscope. To obtain data on the relative frequency of each stage, we examined 1750 transverse sections of testicular seminiferous tubules where 250 sections per animal were identified and counted as in the study of Almeida et al. [36] with modifications. The mean ± standard deviation (SD) and percentages of stages frequency were presented descriptively.

### 2.6. Spermatid Development Observation

Spermatid development in each stage of the SE was observed based on the development of acrosome which includes position and shapes of acrosome in three phases of spermatid development, namely, the Golgi phase, cap phase, and maturation phase as described by Kangawa et al. [33]. The shape and position of spermatid nuclei were observed which started to form the round to elongated spermatid. The data on spermatid development were presented descriptively.

## 3. Results

### 3.1. Seminiferous Epithelium Stages of Aceh Bull

**Figure 1:**
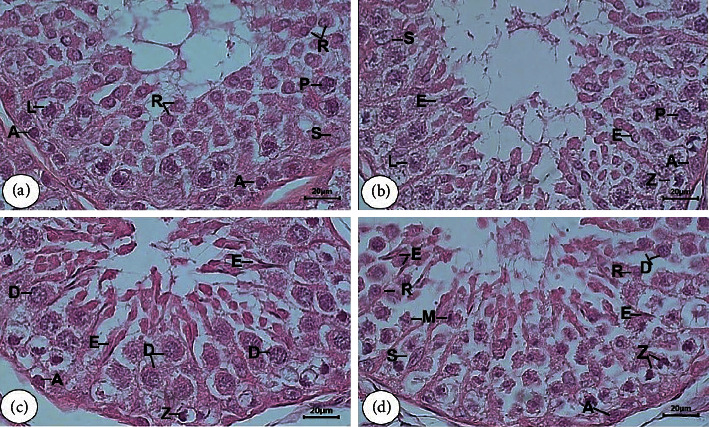
Tubular morphology of stage I to IV of SE in the testicular tissue of Aceh bull. Stage I (a), stage II (b), stage III (c), and stage IV (d). A type spermatogonia (A); leptotene (L), pachytene (P), zygotene (Z), and diplotene (D) of primary spermatocyte; round spermatid (R) and elongated spermatid (E); Sertoli cell (S), and meiosis division (M). H&E staining with bar scale: 20 *μ*m.

**Figure 2:**
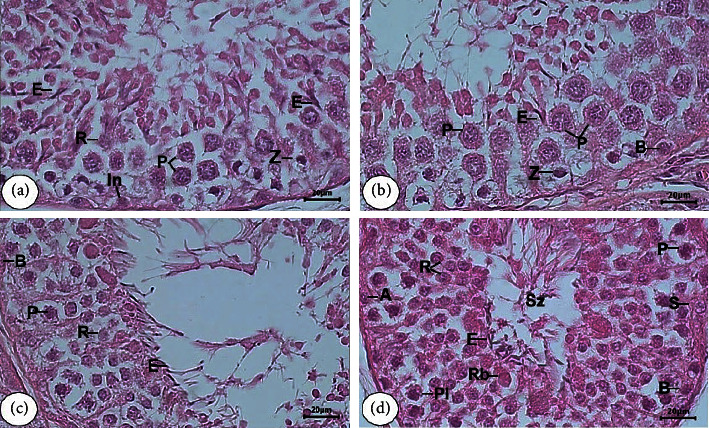
Tubular morphology of stage VI to VIII of SE in the testicular tissue of Aceh bull. Stage V (a), stage VI (b), stage VII (c), and stage VIII (d). A type spermatogonia (A), intermediate type spermatogonia (In), and B type spermatogonia (B); preleptotene (PL), and pachytene (P), and zygotene (Z) of primary spermatocyte; round spermatid (R) and elongated spermatid (E); Sertoli cell (S), residual bodies (Rb), and spermatozoa (Sz). H&E staining, with bar scale: 20 *μ*m.

Through the application of the tubular morphology method, we identified eight stages of development and differentiation of the germinal cells, as well as the appearance of other supporting cells of testicular seminiferous tubules of Aceh bull. Various characteristics of morphology of each stage had also been clearly identified (Figures 1 and 2) and described as follows.

 
*Stage I.* In this stage, we noticed the presence of some germinal cells including spermatogonia A and primary spermatocytes (1^st^ spermatocyte) in the forms of leptotene and pachytene. Furthermore, the round spermatids were clearly found, whereas the elongated spermatids were absent. The Sertoli cells with pale nuclei and conspicuous nucleoli were clearly observable, while their cytoplasm was less clearly identifiable (Figure 1(a)).
 
*Stage II.* The round spermatids began to elongate in this stage. Other germinal cells that appeared in the epithelium tubules were Type A spermatogonium and the 1^st^ spermatocyte in the leptotene, zygotene, and pachytene forms. Additionally, Sertoli cells were also found with conspicuous nuclei scattered between the germinal cells (Figure 1(b)).
 
*Stage III.* The groups of elongated spermatids were detected with the position of their head being toward the basal lamina. Furthermore, zygotene, diplotene spermatocytes, as well as A spermatogonia also appeared at the basal lamina of seminiferous tubules (Figure 1(c)).
 
*Stage IV.* This stage was marked by the main characteristic, namely, the division process of spermatocytes by meiosis resulting in the secondary spermatocyte (the 2^nd^ spermatocyte), which in turn differentiates into round spermatids. Type A spermatogonia, elongated spermatids, and zygotene spermatocytes were also found at the epithelium layer (Figure 1(d)).
 
*Stage V.* The presence of several groups of elongated spermatids located in Sertoli cells crypts of cytoplasmic was identified clearly. In addition to elongated spermatids, the round spermatids, the 1^st^ spermatocytes in forming zygotene and pachytene, A and intermediate (In) spermatogonia at the basal lamina of the tubule, and also Sertoli cells nuclei were discovered in this stage (Figure 2(a)).
 
*Stage VI.* Different from the position in the previous stage, the elongated spermatids began to move away from the basal lamina and become closer to the lumen of the tubule. Other spermatogenic cells in stage V were also present. In this stage, a specific characteristic of pachytene spermatocytes was clearly observed with the largest nuclei compared to these cells found in other stages. Furthermore, B spermatogonia were observable in this stage (Figure 2(b)).
 
*Stage VII.* The elongated spermatids in large numbers were found at the edge of the SE with the tail being in the tubular lumen. Further characteristics were the presence of pachytene spermatocytes with smaller nuclei than those found in stage VI, round spermatids, and also A and B spermatogonia, as well as cytoplasmic of the Sertoli cells with their nuclei (Figure 2(c)).
 
*Stage VIII.* The elongated spermatids in this stage had been separated from SE and entered the tubular lumen as spermatozoa. Round spermatids, pachytene spermatocytes, the nuclei of Sertoli cells, and spermatogonia (A and B) were also clearly seen here (Figure 2(d)).

### 3.2. Seminiferous Epithelium Frequency

Based on eight stages of spermatogenesis observation in the testicular tissue of Aceh cattle, the relative frequency of each stage was obtained in this present study (Table 1). The stage with the highest frequency percentage was stage I (25.48%), followed by stage II (15.38%), stage V (14.97%), stage III (12.92%), stage VII (10.74%), stage VI (10.69), and stage VIII (5.08%). Furthermore, the accumulation of frequency in the premeiotic (stages I, II, and III), meiotic (stage IV), and postmeiotic (stages V, VI, VII, and VIII) phases were 53.78, 4.74, and 41.48%, respectively.

### 3.3. Steps of Spermatid Development

**Figure 3:**
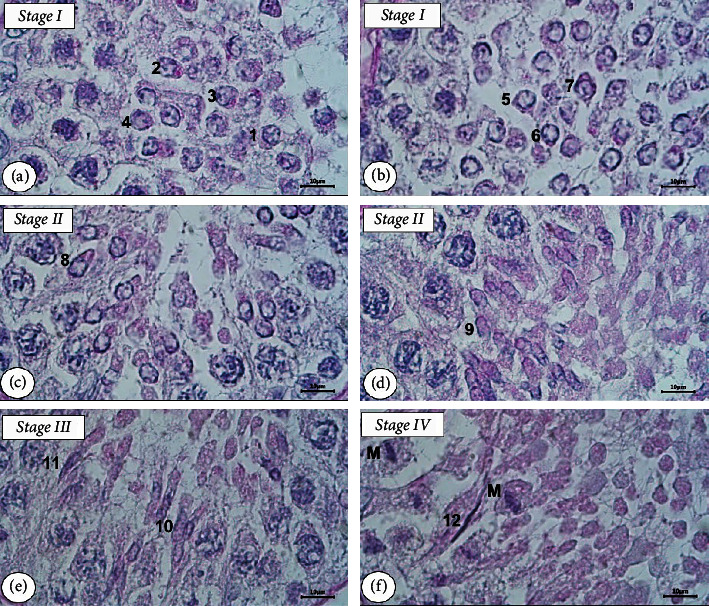
Steps of spermatid development during spermiogenesis of the testis of the Aceh bull. Number 1 to 12 correspond to steps of spermiogenesis. Steps 1 to 7 in stage I (a, b), steps 8 and 9 in stage II (c, d), steps 10 and 11 in stage III (e), step 12 in stage IV (f), and meiosis (M) in stage IV. PAS-H staining with bar scale: 10 *μ*m.

**Figure 4:**
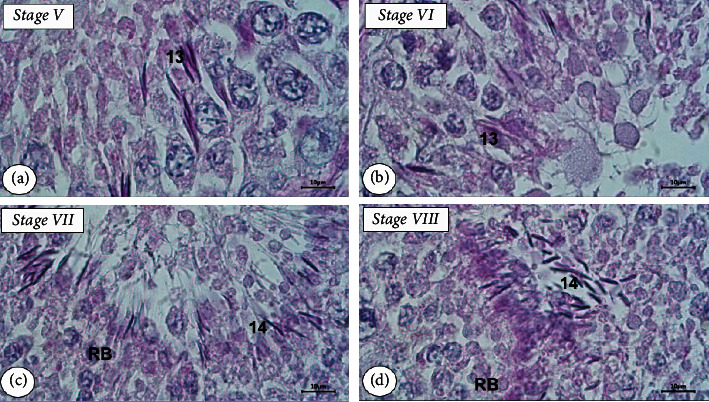
Steps of spermatid development during spermiogenesis of the testis of the Aceh bull. Numbers 13 to 14 in the pictures correspond to steps of spermiogenesis. Step 13 in stages V and VI (a, b), step 14 in stages VII and VIII (c, d), and residual bodies (RB) in stage VII and VIII. PAS-H staining with bar scale: 10 *μ*m.

Having identified the spermatid in the testicular seminiferous tubule of the Aceh bull using PAS-H staining, we found 14 steps of spermatid development in eight stages of SE (Figures 3 and 4). The round spermatids in stage I (step 1) underwent changes in shape, which were characterized by the presence of small granule vesicles (acrosomal granules) in the surface of the nuclear membrane of round spermatid but the granules have not yet attached to the membrane (step 2). Furthermore, the granule vesicles began to approach the nuclear membrane, and indentations were formed on the membrane (step 3) and then moved and firmly attached to the membrane as found in step 4. Still in stage I, round spermatids continued their development marked by the granule beginning to flatten on the cell membrane and forming nearly semicircular acrosome caps on the surface of the membrane (step 5). In step 6 of stage I, the spermatid nucleus is slightly elongated and at the end of the nucleus was found a bulge which was the initial place for the formation of the spermatozoa's tail and its size becomes clearer in step 7.

In stage II of SE, elongated spermatids caused the cells to constrict with the cytoplasm extending caudally (steps 8 and 9). In stage III, the spermatids become more oval (step 10) and conical (step 11) which will develop into the sperm head as found in step 12 of stage IV. The cytoplasm of elongated spermatids started to detach and spermatid tails were formed (step 13) in stages V and VI. The tails of the spermatids were longer and their cytoplasm was separated from the spermatid, namely, residual bodies (step 14) identified in stages VII and VIII. After elongation, the spermatids underwent further maturation which then turned into spermatozoa. The cytoplasm of spermatid in this step will mostly disappear as residual bodies which were further phagocytosed by the cytoplasm of Sertoli cells; hence, spermatozoa would be released from the epithelial layer into the lumen of the seminiferous tubule.

**Figure 5:**
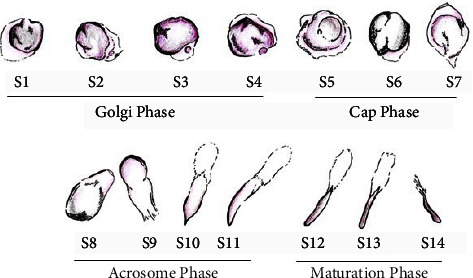
Schematic representation of the four phases of spermatid development during spermiogenesis in the testicular tissue of Aceh bull. Golgi phase, step S1–S4; cap phase, S5–S7; acrosome phase, S8–S11; and maturation phase, S12–S14.

Based on the observation of the development of round spermatids to form spermatozoa, in this present study, we grouped 14 steps of spermatid development into four phases, namely, the Golgi phase, cap phase, acrosome phase, and maturation phase (Figure 5). Steps 1 to 4 were identified as round spermatid differentiation in the Golgi phase, steps 5 to 7 were in the cap phase, steps 8 to 11 in the acrosome phase, and steps 12 to 14 were in the maturation phase.

## 4. Discussion

In the present study, we obtained important data regarding SE stages and steps of spermatid development occurring in the seminiferous tubule of Aceh bull. These findings have not been reported yet in other Indonesian cattle, such as Bali cattle, Madura cattle, and others. According to the tubular morphology observation, eight stages of the SE in the testicular tissue of Aceh bull were clearly identified. Determination of eight stages and identification of cellular composition in each stage of SE in the testicular tissue of Aceh bull is suitable with eight stages in the testicular tissue of goats as reported by França et al. [34]. This method has been applied to obtain the stages of SE during spermatogenesis in testicular tissue in the Javan muntjacs [18]. Based on the eight stages of SE found in testicular tissue of Aceh bull, the development of the spermatids in each stage had been evaluated through the morphological changes of spermatid nuclei and also the position and development of acrosomes in this study. Even though in the previous reports, a cycle of spermatogenesis in brown brocket deer *(Mazama gouazoubira)* [37], Javan muntjacs [18], and other ruminants occurred in eight stages of seminiferous tubule; but in these species, the spermatid development was not described. Observation of each stage of the SE in the testicle of Aceh bull showed different germinal (spermatogenic) cells composition, namely, spermatogonia, spermatocyte, and spermatid which were cleavage and differentiated to produce immature spermatozoa. Although several reports on most ruminants had determined the presence of eight stages of SE, nine stages of the SE were found in rams and described based on the development of the spermatids [20].

In addition to variation in stages, another difference was also found in the relative frequency of each stage, where the highest frequency was found in stage I while the lowest was in stage IV (meiosis stage) as reported in other species, such as goats [31] and Javan muntjacs [18]. The low frequency of the meiotic phase is likely to be related to the short duration of the meiotic division of the 1^st^ spermatocyte in the diplotene form to produce the 2^nd^ spermatocyte. According to Senger [38], the 2^nd^ spermatocyte has a very short lifespan; therefore, these cells are rarely found in the observation of the testicular section.

The relative frequency patterns of all three phases, namely, premeiotic (53.78%), meiotic (4.74%), and postmeiotic (41.48%) of cycle spermatogenesis in the testicular tissue of Aceh bull showed similarity to those goats (49.1%, 10.7%, and 40.2%, respectively) [34] and Javan muntjacs (47.75%, 6.87%, and 45.37%, respectively) [18]. Additionally, our findings showed that the premeiotic phase was more frequently present than the meiotic and postmeiotic phases. There were also some similarities to other species that were reported previously, such as in goats [34], swamp buffalos *(Bubalus bubalis)* [39], and Javan muntjacs [18]. In donkeys and mules [26], however, the postmeiotic phase was more frequently found than the premeiotic and meiotic phases. These differences in the length of the relative frequency of premeiotic, meiotic, and postmeiotic phases in Aceh bull and other ruminants may be related to the lifespan of primary spermatocytes observed in the premeiotic phase, which is longer than the lifespan of other cells (spermatogenic cells) in the testicular seminiferous tubule [38].

An understanding of normal cell composition at each stage seminiferous tubule of the testis can be used as a reference to identify undifferentiated cells in some cases of infertility. This has been applied to the mouse by establishing a quantitative standard composition of seminiferous tubules in each stage of spermatogenesis between normal mice and genetically modified mice deficient [32]. According to Šturm et al. [20], cell associations are typically for the development of each stage that normally comprises spermatogonia, spermatocytes, and spermatids as applied to the identification of stages during a cycle of ram's spermatogenesis. In another report by Lok et al. [40], undifferentiated spermatogonia can be evaluated based on the morphology, proliferation, and differentiation of those cells in the testicular tissue of hamsters and rams. In addition to the SE stages in the testicular tissue of Aceh bull, the value of the relative frequency of each stage of the SE in these cattle can be used as an indicator of the presence of kinetic changes in spermatogenesis that will affect their daily sperm production.

Although there is a large body of research identifying the stages of the SE in the testes using the tubular morphology method using H&E staining, information about the steps of spermatid development using the acrosomal system method detected by PAS-H of histochemical staining has not been widely reported. The development of spermatids was detected in this study based on the eight stages of SE, starting from round to elongated spermatids. The initial process of acrosome formation occurring in the Golgi phase of round spermatids was clearly noticed using the PAS-H staining. The utilization of this staining for acrosome formation observation in each stage of SE during spermatogenesis was successfully applied to the testicular tissue of long-tailed macaques [19] and rams [20]. Acrosome granules that are detected in round spermatids by PAS staining can be used to identify the initial steps of spermatid development, namely, spermiogenesis [41]. The most important morphologic changes during spermiogenesis are formation of the acrosome, condensation of the nuclear chromatin, outgrowth of motile tail of sperm, and loss of excess spermatid material such as cytoplasm, water, and also organelles that are not necessary for the later spermatozoa [42]. The appearance of these acrosomes on the surface of round spermatids was clearly found in this study.

All steps (14 steps) of spermatid differentiation in the testicular tissue of Aceh bull can be grouped into four phases, namely, the Golgi phase, cap phase, acrosome phase, and maturation phase as described in [38, 42, 43]. These four phases of biogenesis acrosome were proposed more than half a century ago by Clermont and Leblond [44] based mainly on their observation using a light microscope on histological preparation of testis that subsequently stained with PAS. In this present study, the Golgi phase of the round spermatids, the initial acrosome formation was found to form the acrosomal vesicles that were evenly distributed on the surface of the round spermatid nuclear membrane as found in steps 2, 3, and 4 of stage I. Furthermore, spermatid differentiation entered the cap phase in step 5 which continued to steps 6 and 7. Meanwhile, in steps 8 and 9, the process of elongation of round spermatids became increasingly clear with a conical acrosome. Both of these steps were in the acrosome phase occurring in stages II and III. Elongated spermatids still were differentiated in stage IV (step 12) that entering the maturation phase where the process continued until step 13 of stages VI and VII and finally differentiation process ended in step 14 of the maturation phase found in stage VIII of the SE.

The results of the present study regarding the 14 steps of spermatid development during the eight stages of SE were also reported in the spermatogenesis of ram's testis by Šturm [20]. However, those steps of spermiogenesis occurred in nine stages, whereas in long-tailed macaque, 12 stages and 14 steps of spermatid differentiation were reported by Dreef et al. [19]. In gerbils, there are 12 stages of SE and 15 steps of spermatid differentiation [30]. The number of stages found in the ram's spermatogenesis is the same as the stages number reported in the testicular tissue of the Guinea cock *(Numida meleagris)*, but the number of steps is slightly different, where in this, cock spermiogenesis occurred in 11 steps [41]. The difference in the number of stages and steps during spermatogenesis among these animal species is species-specific and seems to have a correlation with the number of cycles and duration of spermatogenesis unique to each animal species. Several studies have characterized the spermiogenesis process in mice and have identified 16 steps of spermatid development based on the biogenesis of the acrosome [22].

The data regarding spermatogenesis and spermiogenesis in the testicular tissue of Aceh bull obtained in this study are basic data that complements the previous data regarding correlation between spermatozoa morphometric and testosterone level during the maturation process in the epididymis (caput, corpus, and cauda) which have been reported by Wahyuni et al. [45]. Therefore, further study on the reproductive aspects of Aceh bull is needed to determine the potential of these cattle as producers of good quality of semen to be inseminated in Aceh cows.

## 5. Conclusion

In this study, eight stages of SE in the Aceh bull testes were identified using the tubular morphology method. In addition, 14 steps of spermatid development have also been clearly defined based on the acrosome formation in spermatids.

## Figures and Tables

**Table 1:** The frequency of eight stages of SE in the testicular tissue of Aceh bull.

Stages	Mean ± SD	Frequency (%)
Stage I	63.71 ± 1.98	25.48
Stage II	38.43 ± 2.23	15.38
Stage III	32.29 ± 2.81	12.92
Stage IV	11.86 ± 1.35	4.74
Stage V	37.43 ± 1.81	14.97
Stage VI	26.71 ± 2.36	10.69
Stage VII	26.86 ± 2.41	10.74
Stage VIII	12.71 ± 1.80	5.08
Premeiotic phase (stages I, II, and III)	—	53.78
Meiotic phase (stage IV)	—	4.74
Postmeiotic phase (stages V, VI, VII, and VIII)	—	41.48

## Data Availability

The data used to support the findings of this study are available from the corresponding author upon request.
